# Correction: Attenuated Age-Impact on Systemic Inflammatory Markers in the Presence of a Metabolic Burden

**DOI:** 10.1371/journal.pone.0127137

**Published:** 2015-04-24

**Authors:** The PLOS ONE Staff

## Notice of Republication

This article was republished on April 10, 2015 to correct an error in the first author’s name that was introduced during the typesetting process. Please download this article again to view the correct version. The originally published, uncorrected article and the republished, corrected article are provided here for reference.

## Supporting Information

S1 File Originally published, uncorrected article. (PDF)

S2 File Republished corrected article. (PDF)
