# Supplementary material for: Attenuated Age-Impact on Systemic Inflammatory Markers in the Presence of a Metabolic Burden
Source: PLoS One. 2015 Mar 27;10(3):e0121947. doi: 10.1371/journal.pone.0121947 (PMC4376898; doi:10.1371/journal.pone.0121947)
Supplement: S1 Table — (DOCX) [file pone.0121947.s001.docx]

**Supplemental Material**

**Table S1. Pearson’s partial (adjusted for gender) correlation coefficients between age and inflammatory markers in subjects with and without MetS* across race.**

| **Inflammatory markers** | **MetS(-)**  (n=219) | | **MetS(+)**  (n=309) | |
| --- | --- | --- | --- | --- |
|  | r | *P* | r | *P* |
| **Caucasians** |  |  |  |  |
| *Systemic* |  |  |  |  |
| CRP (mg/l) | 0.248 | 0.015 | 0.038 | NS |
| Fibrinogen (mg/dl) | 0.373 | <0.001 | 0.237 | 0.001 |
| SAA (mg/l) | 0.389 | <0.001 | -0.007 | NS |
| Composite z-score, systemic | 0.291 | 0.004 | 0.047 | NS |
| *Vascular* |  |  |  |  |
| Lp-PLA_2_ mass (ng/ml) | 0.021 | NS | 0.251 | 0.001 |
| Lp-PLA_2_ activity (nmol/min/ml) | -0.026 | NS | 0.046 | NS |
| PTX-3 (ng/ml) | 0.090 | NS | 0.077 | NS |
| Composite z-score, vascular | -0.003 | NS | 0.045 | NS |
| **African Americans** |  |  |  |  |
| *Systemic* |  |  |  |  |
| CRP (mg/l) | 0.206 | 0.035 | 0.021 | NS |
| Fibrinogen (mg/dl) | 0.300 | 0.002 | 0.154 | NS |
| SAA (mg/l) | 0.290 | 0.003 | -0.041 | NS |
| Composite z-score, systemic | 0.271 | 0.005 | 0.023 | NS |
| *Vascular* |  |  |  |  |
| Lp-PLA_2_ mass (ng/ml) | 0.043 | NS | -0.015 | NS |
| Lp-PLA_2_ activity (nmol/min/ml) | 0.103 | NS | -0.015 | NS |
| PTX-3 (ng/ml) | -0.049 | NS | 0.165 | NS |
| Composite z-score, vascular | 0.053 | NS | -0.034 | NS |

Data for CRP, SAA, Lp-PLA_2_ and PTX-3 were logarithmically transformed to normalize the distribution of marker values before statistical analyses. CRP indicates C-reactive protein; SAA, serum amyloid-A; Lp-PLA_2_, lipoprotein associated phospholipase A_2_, PTX-3, pentraxin-3; NS, not significant. *Data based on n=304 Caucasians and n=224 African American subjects.
