# Supplementary figures and images for: Attenuated Age-Impact on Systemic Inflammatory Markers in the Presence of a Metabolic Burden
Source: PLoS One. 2015 Mar 27;10(3):e0121947. doi: 10.1371/journal.pone.0121947 (PMC4376898; doi:10.1371/journal.pone.0121947)

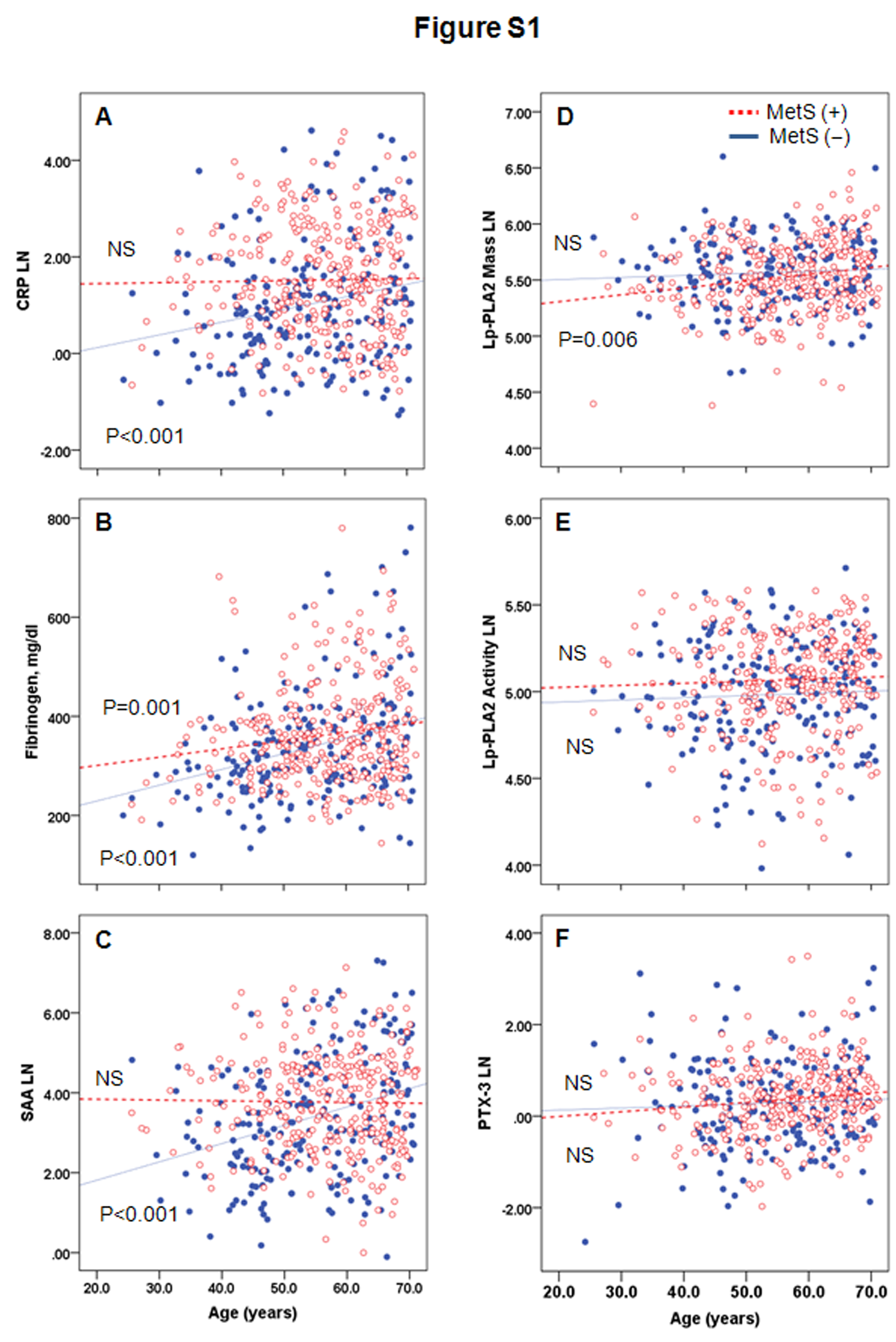

Supplement: S1 Fig — A, C-reactive protein (CRP). B, Fibrinogen. C, Serum amyloid-A (SAA). D, Lipoprotein associated phospholipase A2 (Lp-PLA2) mass; E, Lp-PLA2 activity. F: Pentraxin-3 (PTX-3). LN indicates logarithmically transformed variables. Lines represent unadjusted linear regression slopes. (TIF) [file pone.0121947.s002.tif]
