# Supplementary material for: Correction: Attenuated Age-Impact on Systemic Inflammatory Markers in the Presence of a Metabolic Burden
Source: PLoS One. 2015 Apr 24;10(4):e0127137. doi: 10.1371/journal.pone.0127137 (PMC4409308; doi:10.1371/journal.pone.0127137)
Supplement: S2 File — (PDF) [file pone.0127137.s002.pdf]

RESEARCH ARTICLE

Attenuated Age-Impact on Systemic Inflammatory Markers in the Presence of a Metabolic Burden

Erdembileg Anuurad[1], Annie Mirsoian[2], Byambaa Enkhmaa[1], Wei Zhang[1], Laurel A. Beckett[3], William J. Murphy[1,2], Lars F. Berglund[1,4]*

1 Department of Medicine, University of California Davis, Davis, CA, United States of America,
2 Department of Dermatology University of California Davis, Davis, CA, United States of America,
3 Department of Public Health Sciences, University of California Davis, Davis, CA, United States of America,
4 The VA Northern California Health Care System, Sacramento, CA, United States of America

* lars.berglund@ucdmc.ucdavis.edu

CrossMark
click for updates

OPEN ACCESS

Citation: Anuurad E, Mirsoian A, Enkhmaa B, Zhang W, Beckett LA, Murphy WJ, et al. (2015) Attenuated Age-Impact on Systemic Inflammatory Markers in the Presence of a Metabolic Burden. PLoS ONE 10(3): e0121947. doi:10.1371/journal.pone.0121947

Academic Editor: Ana Mora, University of Pittsburgh, UNITED STATES

Received: August 8, 2014

Accepted: February 16, 2015

Published: March 27, 2015

Copyright: © 2015 Erdembileg et al. This is an open access article distributed under the terms of the Creative Commons Attribution License, which permits unrestricted use, distribution, and reproduction in any medium, provided the original author and source are credited.

Data Availability Statement: All relevant data are within the paper and its Supporting Information files.

Funding: This work was supported by National Institutes of Health HL62705, TR000002, CA095572, RR024144, 2K12HD051958; and American Heart Association 14CRP17930014. The sponsors provided funding in response to grant applications but had no role in design of the study, data collection or manuscript preparation.

Competing Interests: The authors have declared that no competing interests exist.

Abstract

Background

The overall burden of chronic disease, inflammation and cardiovascular risk increases with age. Whether the relationship between age and inflammation is impacted by presence of an adverse metabolic burden is not known.

Methods

We determined inflammatory markers in humans (336 Caucasians and 224 African Americans) and in mice, representing a spectrum of age, weight and metabolic burden.

Results

In humans, levels of inflammatory markers increased significantly with age in subjects without the metabolic syndrome, (P=0.009 and P=0.037 for C-reactive protein, $P<0.001$ and P=0.001 for fibrinogen, $P<0.001$ and P=0.005 for serum amyloid-A, for Caucasians and African Americans, respectively). In contrast, trend patterns of inflammatory markers did not change significantly with age in subjects with metabolic syndrome in either ethnic group, except for fibrinogen in Caucasians. A composite z-score for systemic inflammation increased significantly with age in subjects without metabolic syndrome (P=0.004 and $P<0.006$ for Caucasians and African Americans, respectively) but not in subjects with metabolic syndrome (P=0.009 for difference in age trend between metabolic syndrome and non-metabolic syndrome). In contrast, no similar age trend was found in vascular inflammation. The findings in humans were paralleled by results in mice as serum amyloid-A levels increased across age (range 2-15 months, $P<0.01$) and were higher in ob/ob mice compared to control mice ($P<0.001$).

Conclusions

Presence of a metabolic challenge in mice and humans influences levels of inflammatory markers over a wide age range. Our results underscore that already at a young age,

presence of a metabolic burden enhances inflammation to a level that appears to be similar to that of decades older people without metabolic syndrome.

Introduction

Inflammation is considered as a critical factor for the development of cardiovascular disease (CVD)[1]. The atherosclerotic process is hallmarked by the infiltration of macrophages and T cells at the sites of lipid deposition causing the release of cytokines and other bioactive molecules that lead to further infiltration of immune cells. Together, this inflammatory process perpetuates the formation of a fibrous cap composed mostly of collagen boarded by infiltrating immune cells that ultimately allows for plaque growth and ischemia [2]. Therefore, the development of atherosclerotic lesions in the vascular wall is the net result of many complex processes where multiple proinflammatory risk factors contribute by influencing individual pathophysiological steps [3].

The predictive role of systemic inflammatory markers in CVD is well established, and we and others have demonstrated that vascular inflammation is also associated with CVD risk [4]. However, there is a paucity of data indicating to what extent the inflammatory process is accelerated by the presence of a disadvantageous metabolic setting, such as for example the presence of metabolic syndrome (MetS) [5]. Many of the components of MetS, such as hypertriglyceridemia, low high density lipoprotein (HDL) cholesterol levels, hypertension, abdominal obesity, and increased fasting glucose levels are present early in life resulting in a lifelong exposure. Much attention has been concentrated on the lifelong relationship between MetS components and the immune system with current research indicating both as the developmental origins of chronic disease [6]. With an increasing sedentary lifestyle and increasingly high rates of obesity, exceeding 30% in the US population for most age and gender groups [7], such conditions are common and the age-adjusted prevalence of MetS is presently 34% [8].

Although traditional cardiovascular risk factors are useful for predicting cardiovascular events in younger populations, their predictive value decreases with age [9]. Therefore, one could speculate that presence of an additional inflammatory component, such as MetS, may play a more substantial role in the pathogenesis of CVD in older compared to younger individuals. However, to what extent an inflammatory contribution to CVD development might be modulated by age in the absence or presence of a metabolic burden is not known. To test the relationship between metabolic burden, age, and inflammation and whether a similar pattern is seen across species, we undertook studies in both humans and mice. We hypothesized that the degree of an age-associated increase in inflammatory markers would depend on the absence or presence of a metabolic burden, and that this would be present in both humans and mice. Data obtained through this study has the potential to inform an understanding of CVD risk throughout the process of aging and to build a foundation to develop strategies for early risk intervention.

Materials and Methods

Human Subjects

Subjects were recruited from a patient population scheduled for diagnostic coronary arteriography either at Harlem Hospital Center in New York City or at the Mary Imogene Bassett Hospital in Cooperstown, NY. The study design including exclusion and inclusion criteria has

been described previously [10,11]. Briefly, a total of 648 patients, self-identified as Caucasians (n = 344), African American (n = 232) or Other (n = 72) were enrolled. Exclusion criteria for this study included the use of lipid-lowering drugs, as well as hormone replacement therapies. The present report is based on findings in 560 subjects (336 Caucasians, 224 African Americans); 16 subjects were excluded due to incomplete data. The study was approved by the Institutional Review Boards at Harlem Hospital, the Mary Imogene Bassett Hospital, Columbia University College of Physicians and Surgeons, and University of California Davis, and informed consent was obtained from all subjects.

Coronary Angiography

The coronary angiograms were read by 2 experienced readers blinded to patient identity, the clinical diagnosis, and laboratory results. The readers recorded the location and extent of luminal narrowing for 15 segments of the major coronary arteries [12]. In the present study, patients were classified as having CAD if a stenosis of $\geq$50% was found in at least one of the segments. Patients without CAD were defined as having <50% stenosis in all of the segments. A composite cardiovascular score (0–75) was calculated based on determination of presence of stenosis on a scale of 0–5 of the 15 predetermined coronary artery segments.

Human Clinical and Biochemical Assessment

Blood pressure was measured with a random-zero mercury sphygmomanometer. Waist circumference was calculated as the average of 2 measurements taken after inspiration and expiration at the midpoint between the lowest rib and iliac crest. Participants were asked to fast for 12 hours, and blood samples were drawn approximately 2 to 4 hours before the catheterization procedure. Serum and plasma samples were separated and stored at -80°C prior to analysis. Concentrations of total and HDL cholesterol and glucose (Roche, Sommerville, NJ) were determined using standard enzymatic procedures [13,14]. HDL cholesterol levels were measured after precipitation of apoB-containing lipoproteins with dextran sulfate [15], and LDL cholesterol levels were calculated with the formula of Friedewald *et al* [16]. High-sensitivity C-reactive protein (CRP) levels were measured using an enzyme-linked immunoabsorbent assay (ELISA), standardized according to the World Health Organization First International Reference Standard [17,18]; CV 8.9%. Fibrinogen levels were measured by the clot-rate method of Clauss [19]; CV 3.0%. Insulin levels were assessed using a Coat-A-Count RIA kit (DPC Diagnostic Products Co, Los Angeles, CA). Homeostasis model assessment—insulin resistance (HOMA-IR) was calculated using the updated model available from the Oxford Centre for Endocrinology and Diabetes [20]. Pentraxin-3 (PTX-3) was measured by PTX-3 (human) Detection Set from Alexis Biochemicals (Axxora, LLC); CV 10.2%. Plasma serum amyloid-A (SAA) concentrations were measured by ELISA using a commercially available kit (Invitrogen, Inc., Carlsbad, CA) [21]; CV 5.4–10.3%. Lipoprotein-associated phospholipase A_2 (Lp-PLA_2) mass was assayed using a microplate-based ELISA; CV 4.8–7.2%, and Lp-PLA_2 activity was measured with a colorimetric activity method (diaDexus, Inc., South San Francisco, CA) [22–24]; CV 6.5–7.8%. All biochemical assessments were made in duplicate.

We defined the MetS using the revised NCEP-ATP III criteria [25] as having three or more of the following parameters: fasting plasma glucose $\geq$100 mg/dl, serum triglycerides $\geq$150 mg/dl, serum HDL cholesterol <40 mg/dl for men and <50 mg/dl for women, blood pressure $\geq$130/85 mmHg, or undergoing hypertension treatment, or waist circumference of more than 102 cm for men and 88 cm for women.

Animal Models

Female young (2 months), middle-aged (12 months), and aged (15–18 months) C57BL/6 mice were purchased from Jackson Laboratory (Bar Harbor, ME). For experiments using middle-age and aged mice, mice were purchased from Jackson Laboratory between the ages of 6–9 months and further aged at the University of California Davis's Sacramento campus. Additionally, young (2 months) obese mice congenic for the spontaneous leptin mutation (B6.V-Lep$^{ob/ob}$/J) were also purchased from Jackson Laboratory (Bar Harbor, ME). Mice were housed under specific pathogen-free conditions, and all mouse studies were conducted with the approval of the UC Davis Institutional Animal Care and Use Committee.

For studies involving the use of aged ad libitum fed and aged calorie restricted mice, age-matched mice were purchased through the NIA Aged Colony (Bethesda, MD). Calorie-restricted cohorts were fed the NIH31-fortified diet. In accordance with the NIA's Aged Colony guidelines, the NIA achieves calorie-restriction by first placing the mice at a 10% calorie restriction, initiated at 14 weeks of age. The restriction is then increased to 25% at 15 weeks of age, and to 40% at 16 weeks of age. The mice are maintained at 40% calorie restriction throughout life thereafter. Age-matched ad libitum fed aged mice were also purchased through the NIA colony to serve as controls.

Murine Assessment of SAA Levels

Murine serum levels of SAA were quantified using a mouse SAA ELISA kit according to the manufacturer's protocols; CV 2.7–5.6% (Immunology Consultants Laboratory, Portland, OR). Briefly, serum was obtained through tail bleeds of resting mice. Eight serial dilutions of standards of known concentration were performed and plated in accordance to manufacturer's instructions. Serum samples were diluted 1:1000 and plated in duplicate into pre-designated wells. Samples and standards were incubated in pre-coated wells at room temperature for 60 minutes, washed four times with Wash Solution prior to addition of 100 μl of Enzyme-Antibody Conjugate. The wells were incubated at room temperature for 60 minutes in the dark and washed four times as described above. 100 μl of TMB substrate solution was then added to each well, incubated at room temperature for precisely 10 minutes, and the reaction stopped by addition of 100 μl of Stop Solution. Colorimetric determination of SAA levels was performed by reading of absorbance of each well at 450 nm on a plate reader (VERSAma turntable plate reader).

Murine Adiposity Imaging

Magnetic resonance imaging (MRI) images for mouse data were acquired in collaboration with the Center for Molecular and Genomic Imaging (CMGI) Facility at the University of California, Davis. Mice were imaged using a Bruker BioSpec 70/30 7T (Bruker Biospin, Ettlingen, Germany) horizontal bore system. Animals were anesthetized with 2% isofluorane and maintained with 1–2% isoflurane throughout imaging with a nose cone fitted for inhalation. Mice were kept at normal body temperature using circulating warm air. Mice were inserted into the scanner in a head-first prone orientation. Spin-echo T1-weighted images were obtained through the mouse body (neck-to-base of the tail) using a 72 mm linear volume coil. Scan sequence parameters were the following: TR 1000 ms, TE 15 ms and 2 averages. The field of view was 7.7 × 3.85 x 2.0 cm, with a matrix size of 256 x 128 x 40. The corresponding voxel size was 0.3 × 0.3 × 0.5 mm. Images were acquired using ParaVision 5.0 software. After acquisition, images were transferred into Invenon Research Workplace 4.0 software (Siemens Preclinical) allowing fat to be displayed with high intensity (white).

Statistical Analysis

For the mouse studies, statistical analysis was performed using Prism software (GraphPad Software Inc.). Data were expressed as mean ± SE. For analysis of three or more groups, the one-way ANOVA test was performed with the Bonferroni *post-hoc* test. Analysis of differences between two test groups was performed using the Student's *t*-test. A minimum of three mice per group was used for serum assays. For human studies, analysis of data was done with SPSS statistical analysis software (SPSS Inc, Chicago, IL). Results were expressed as means ± standard error of the mean (SEM). Levels of CRP, SAA, PTX-3, triglycerides, insulin, HOMA-IR, Lp-PLA_2 mass, Lp-PLA_2 activity and the cardiovascular score were logarithmically transformed to achieve normality for statistical analysis. Proportions were compared between groups using χ^2 test or Fisher's exact test as appropriate. General linear measurement analyses were used for anthropometric, metabolic and clinical parameters after adjustment for age and sex. Sex-adjusted Pearson's partial correlation coefficients were calculated for age and inflammatory markers across ethnicities. To construct a composite score of multiple markers, we first calculated a z-score for each inflammatory marker [$z = (x - \bar{x})/SD$, where x is an individual marker value, $\bar{x}$ is the mean marker value, and SD is the standard deviation of marker values]. Using the individual z-scores, we next calculated a composite z-score for systemic inflammation [i.e., z-score (systemic) = Average (z-CRP, z-fibrinogen, z-SAA)], and for vascular inflammation [z-score (vascular) = Average (0.5 z-Lp-PLA_2 mass, 0.5 z-Lp-PLA_2 activity, z-PTX-3)], as described previously [4]. As we used Lp-PLA_2 mass and activity to calculate a composite z-score for vascular inflammation, these two parameters were given a coefficient of 0.5 in the formula. Multivariate linear regression analysis was performed to explore the independent association of age with the composite z-scores adjusted for confounding variables. An interaction term for age by presence of MetS allowed a formal test of the difference in age trends for MetS compared to non-MetS patients, and further calculations based on this model addressed the trend for MetS and the differences at both younger and older ages. Model assumptions of linearity and normality and homoscedasticity of residuals were validated both analytically and graphically. Two-tailed *p* values less than 0.05 were considered statistically significant.

Results

Aging and Obesity Result in Increased Circulating Acute-Phase Proteins in Normal Mice

We and others have previously shown that circulating levels of acute phase proteins are correlated with increased risk for CVD [1,4,26,27]. We first sought to examine the impact of aging upon increased circulating levels of acute phase proteins within mice of varying ages. Because of species differences between human and mice, serum amyloid A (SAA), a major acute phase protein indicative of inflammatory responses in mice that is analogous to CRP in humans, was measured among mice representing increasing ages. Young (2 months), middle aged (12 months), and aged (15–18 months) mice were assessed for SAA levels at a resting state. Accordingly, SAA levels increased gradually with age from two to 18 months resulting in a significant correlation between SAA levels and aging (Fig. 1A).

Aside from age, it was observed that aged mice inherently display increased body mass in comparison to young lean mice, yet weigh less than young obese (*ob/ob*) mice (Fig. 1B). It is well documented that aging is associated with changes in body mass composition, hallmarked by the loss of lean muscle mass and increase in central, visceral, adiposity [28–30]. Therefore, we questioned if the increased presence of adipose tissue affected circulating levels of inflammatory markers with aging, thereby increasing risk for CVD. To determine adiposity and fat

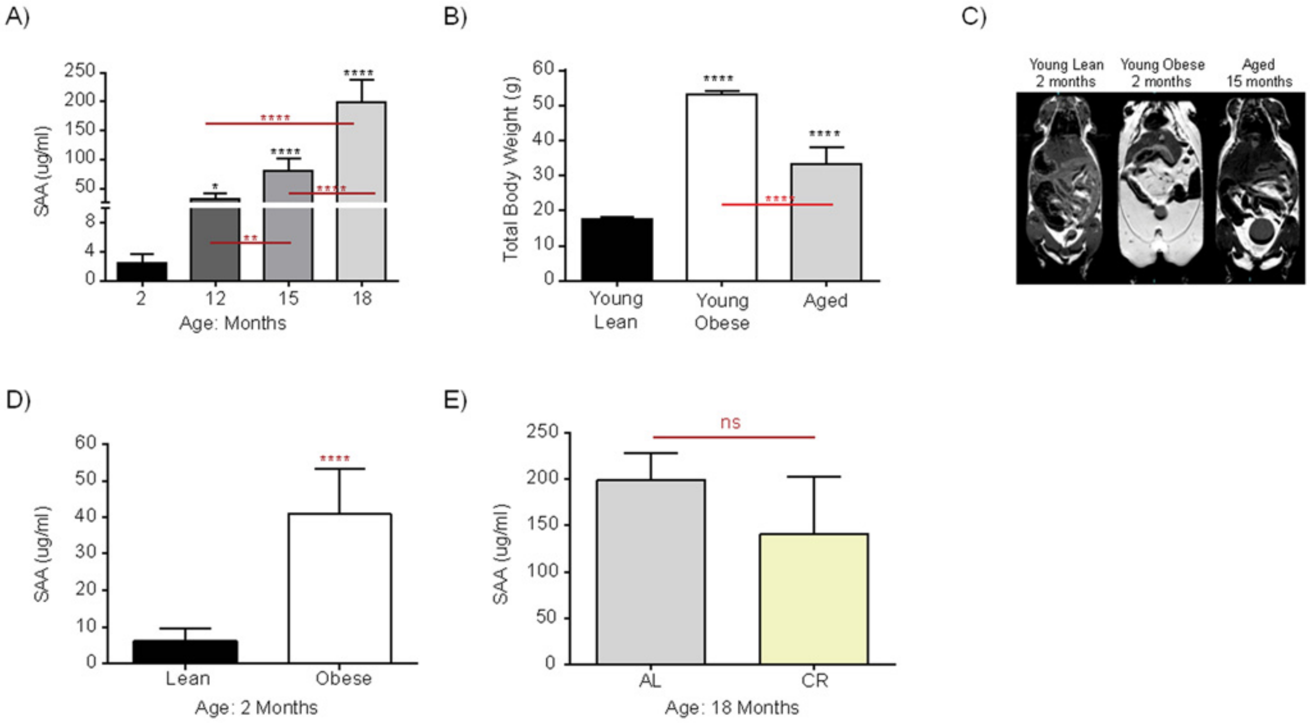

Fig 1. Serum SAA levels in C57BL/6 mice of 2, 12, 15, and 18 months of age (**A**). Total body weights were assessed for young lean, young obese (ob/ob), and aged mice (**B**). MRI images demonstrating distribution of fat deposits in young lean, young obese and aged mice (**C**). Serum SAA levels in young lean and age-matched young obese (2 months old) (**D**), and age-matched aged ad libitum-fed and calorie-restricted mice (18 months of age) (**E**). N = 3–13, *P<0.05, **P<0.01, ***P<0.001, ****P<0.0001.

doi:10.1371/journal.pone.0121947.g001

deposition within mice, we employed the usage of MRI. For comparison to an obesity model, young (2 months, *ob/ob*) congenic obese mice (B6.Cg-Lep$^{ob/ob}$) were included and compared to young age-matched lean and aged (15 months) mice. MRI imaging demonstrated aged mice to exhibit greater visceral adiposity in comparison to their lean young counterparts, with obese mice exhibiting the greatest amount of fat deposits (Fig. 1C).

To dissect the role of metabolic dysregulation on increasing SAA levels, we compared serum levels of resting young lean and young obese mice (*ob/ob*). *Ob/ob* mice are congenic for the mutation in the OB gene resulting in loss of leptin hormone and exhibit symptoms of MetS including visceral obesity, a diabetes-like syndrome of hyperglycemia, elevated plasma insulin, and increased circulating LDL and VLDL cholesterol levels (http://jaxmice.jax.org/strain/000632.html#pheno). Age-matched young *ob/ob* mice demonstrated a significant increase in SAA levels in comparison to their age-matched young lean counterparts (Fig. 1D). This data demonstrates that increased adiposity may play a role in inducing increased levels of SAA.

To further determine if age alone, in the absence of adiposity, is crucial for the increased SAA levels observed in Fig. 1A, we measured serum levels within age-matched (18 month old) aged calorie-restricted (CR) mice in comparison to aged mice following an ad libitum diet (AL). Notably, when comparing aged AL fed mice with age-matched, CR mice, a lower body fat content did not result in any significant differences in SAA levels (Fig. 1E). These results indicate that an increased metabolic burden as well as age contributes to the elevation of inflammatory markers.

Human Studies Parallel Observations in Mouse Model

Based upon our data observed in the mouse model, we next sought to examine the relationship of age and inflammatory markers in the presence of metabolic burden using patient derived samples. Clinical characteristics of study subjects with and without MetS are shown in Table 1. As seen in the table, subjects with MetS were slightly older and more obese, had significantly higher levels blood pressure, as compared to those without MetS. Levels of CRP, fibrinogen, Lp-PLA_2 activity and PTX-3 were higher in subjects with MetS compared to those without MetS, while Lp-PLA_2 mass levels were significantly lower in the former group.

Table 1. Clinical characteristics of study subjects (adjusted for race and sex).

**Characteristics**	**Without MetS** (n = 219)	**With MetS** (n = 309)	***P*-value**
Hypertension (%)	96 (44%)	238 (77%)	<0.001
Postmenopausal (%)	46 (21%)	105 (34%)	0.001
Smoking (%)	86 (39%)	88 (28%)	0.009
Alcohol (%)	94 (43%)	105 (34%)	0.036
Anthropometric			
Age (yrs)	54.6±0.7	56.5±0.6	0.021
BMI (kg/m^2)	26.0±0.4	31.5±0.3	<0.001
Waist circumference (cm)	91.5±1.0	108.0±0.9	<0.001
Systolic blood pressure (mm Hg)	121±1	130±1	<0.001
Diastolic blood pressure (mm Hg)	74±1	77±1	<0.001
Inflammatory markers			
Systemic			
CRP (mg/l)	2.4 (1.1–5.2)	4.2 (1.9–11.1)	0.036
SAA (mg/l)	25 (10–92)	45 (18–102)	NS
Fibrinogen (mg/dl)	333±7	366±6	<0.001
Vascular			
Lp-PLA_2 mass (ng/ml)	259 (213–323)	249 (209–312)	<0.001
Lp-PLA_2 activity (nmol/min/ml)	151 (119–181)	163 (137–194)	<0.001
PTX-3 (ng/ml)	1.28 (0.72–2.57)	1.42 (0.93–2.25)	0.144
Metabolic			
Total cholesterol (mg/dl)	193±3	200±2	0.006
LDL cholesterol (mg/dl)	119±3	127±2	NS
HDL cholesterol (mg/dl)	51±1	39±1	<0.001
Triglyceride (mg/dl)	99 (75–130)	159 (116–216)	<0.001
Glucose (mg/dl)	107±4	140±3	<0.001
Insulin (μU/ml)	10.6 (7.2–16.1)	17.2 (11.9–29.7)	NS
HOMA-IR	1.4 (0.9–2.1)	2.3 (1.6–4.0)	<0.001
ApoA-I (mg/dl)	133±2	120±1	<0.001
ApoB (mg/dl)	125±3	141±2	<0.001
Cardiovascular diseaseComposite cardiovascular score	5.7 (0.0–20.0)	15.7 (4.2–30.0)	<0.001

Data are means ± SEM or for non-normally distributed variables as median (interquartile range). General linear measurement analyses were used for anthropometric, metabolic and clinical parameters after adjustment for age and sex. Values for triglyceride, insulin, CRP, SAA, Lp-PLA_2, PTX-3, HOMA-IR and composite cardiovascular score were logarithmically transformed to normalize the distribution before statistical analyses. CRP indicates C-reactive protein; SAA, serum amyloid-A; Lp-PLA_2, lipoprotein associated phospholipase A_2, PTX-3, pentraxin-3; LDL, low density lipoprotein; HDL, high density lipoprotein; HOMA-IR, Homeostasis model assessment—insulin resistance; ApoA-I, apolipoprotein A-I; ApoB, apolipoprotein B; NS, not significant.

doi:10.1371/journal.pone.0121947.t001

To explore the relationship between metabolic burden, inflammation, and age we used two approaches based on body mass index (BMI) or presence of MetS. We first analyzed the correlation of inflammatory markers with age for three different BMI groups, representing normal weight (BMI $\leq$25), overweight (BMI 25–29.9) and obese (BMI $\geq$30) subjects. As see in Table 2, there was a significant correlation between the three systemic inflammatory markers and age in the normal weight group, while only fibrinogen was significantly correlated with age in the overweight and obese subjects. Further, the z-score, representing a combination of the systemic markers was associated with age only for the normal weight group. In contrast, the only significant correlation between vascular inflammatory markers and age was seen for Lp-PLA$_2$ mass among obese subjects. Notably, the z-score for vascular inflammation was not associated with age in any BMI group.

When using presence or absence of the MetS to illustrate the effects of metabolic burden, we noted that all three systemic inflammatory markers as well as the z-score for systemic inflammation were robustly associated with age among subjects without the MetS (Table 3). In contrast, no correlations with age were observed for vascular inflammatory markers as well as the z-score for vascular inflammation among subjects without the MetS. In parallel with our findings across BMI groups, only fibrinogen levels and Lp-PLA$_2$ mass were associated with age among subjects with the MetS. The trend pattern of each individual inflammatory marker with age is shown in S1 Fig. For the composite z-scores, no correlation was seen for either systemic or vascular markers with age among subjects with the MetS. As seen in S1 Table, the findings with regard to systemic inflammatory markers were similar for African Americans and Caucasians without the MetS. For subjects with the MetS, significant associations between fibrinogen or Lp-PLA$_2$ with age was only seen among Caucasians. Taken together this data shows that systemic, but not vascular inflammatory markers were associated age in subject without MetS.

Multiple regression models, adjusted for race, sex, BMI, LDL-C, HDL-C and triglyceride, confirmed the relationships between age and the composite z-score for systemic and vascular inflammation in subjects with and without the MetS (Table 4). Systemic inflammation on average was 0.3 standard deviation (SD) higher with each decade older in subjects without MetS (P<0.001). Subjects with MetS at age 30 already had mean systemic inflammation dramatically higher than age-comparable counterparts without MetS (P = 0.004) and as high as those

Table 2. Pearson's partial (adjusted for race and sex) correlation coefficients between age and inflammatory markers across BMI groups *.

Inflammatory markers	**BMI $\leq$25** (n = 140)		**BMI 25–29.9** (n = 191)		**BMI >30** (n = 218)	
	**r**	***P***	**r**	***P***	**r**	***P***
Systemic						
CRP (mg/l)	0.262	0.002	0.100	NS	0.101	NS
Fibrinogen (mg/dl)	0.249	0.004	0.330	<0.001	0.215	0.002
SAA (mg/l)	0.317	<0.001	0.088	NS	0.121	NS
Composite z-score, systemic	0.180	0.038	0.144	NS	0.132	NS
Vascular						
Lp-PLA$_2$ mass (ng/ml)	0.007	NS	0.111	NS	0.148	0.032
Lp-PLA$_2$ activity (nmol/min/ml)	-0.037	NS	0.085	NS	0.030	NS
PTX-3 (ng/ml)	-0.048	NS	0.085	NS	0.037	NS
Composite z-score, vascular	-0.002	NS	0.041	NS	-0.002	NS

Data for CRP, SAA, Lp-PLA$_2$ and PTX-3 were logarithmically transformed to normalize the distribution of marker values before statistical analyses. CRP indicates C-reactive protein; SAA, serum amyloid-A; Lp-PLA$_2$, lipoprotein associated phospholipase A$_2$, PTX-3, pentraxin-3; NS, not significant.

doi:10.1371/journal.pone.0121947.t002

Table 3. Pearson's partial (adjusted for race and sex) correlation coefficients between age and inflammatory markers in subjects with and without MetS.

Inflammatory markers	Without MetS (n = 219)		With MetS (n = 309)	
	r	*P*	r	*P*
Systemic				
CRP (mg/l)	0.226	0.001	0.035	NS
Fibrinogen (mg/dl)	0.330	<0.001	0.201	0.001
SAA (mg/l)	0.333	<0.001	-0.016	NS
Composite z-score, systemic	0.232	0.001	0.022	NS
Vascular				
Lp-PLA_2 mass (ng/ml)	0.031	NS	0.161	0.006
Lp-PLA_2 activity (nmol/min/ml)	0.038	NS	0.021	NS
PTX-3 (ng/ml)	0.017	NS	0.110	NS
Composite z-score, vascular	0.028	NS	0.006	NS

Data for CRP, SAA, Lp-PLA_2 and PTX-3 were logarithmically transformed to normalize the distribution of marker values before statistical analyses. CRP indicates C-reactive protein; SAA, serum amyloid-A; Lp-PLA_2, lipoprotein associated phospholipase A_2, PTX-3, pentraxin-3; NS, not significant.

doi:10.1371/journal.pone.0121947.t003

counterparts who were three decades older, as illustrated in unadjusted linear regression lines (Fig. 2A). After adjustment for covariates, subjects with MetS showed only a slight increase in systemic inflammation with age of 0.011 per year (from Table 4, the slope for MetS (-) plus the difference in slopes, or 0.030–0.019, *P* = 0.03), significantly flatter than for non-MetS subjects (*P* = 0.009). Indeed, at by age 70, the MetS subjects no longer had significantly greater systemic inflammation than their age-comparable counterparts without MetS (difference 0.16, *P*>0.5). Vascular inflammatory markers showed neither a significant trend with age, nor any difference in patterns between MetS and non-MetS subjects (Table 4, Fig. 2B). Patterns were similar within both African Americans and Caucasians (Fig. 2C, 2D). Collectively, these results suggest a

Table 4. Multiple regression analysis of systemic and vascular z-scores with age, MetS and other cardiovascular risk factors in Caucasians and African Americans adjusted for confounders *.

Model	Independent variables	R^2	*F*	β	*P*
Model 1					
(systemic)		0.169	11.4		<0.001
	Age_30			0.030	<0.001
	MetS			0.602	0.004
	Age_30 × MetS			-0.019	0.009
Model 2					
(vascular)		0.191	13.1		<0.001
	Age_30			0.004	NS
	MetS			-0.092	NS
	Age_30 × MetS			0.002	NS

* Adjusted for race, sex, BMI, LDL cholesterol, HDL cholesterol and triglycerides.
A young healthy reference person for regression estimate was defined as age 30, non-MetS, Caucasian, male. All parameter estimates are estimated the differences from outcome from reference person. Age_30 refers to trends with age compared to 30 years old person. Interactions with age estimate how the MetS modifies the trend with age.

doi:10.1371/journal.pone.0121947.t004

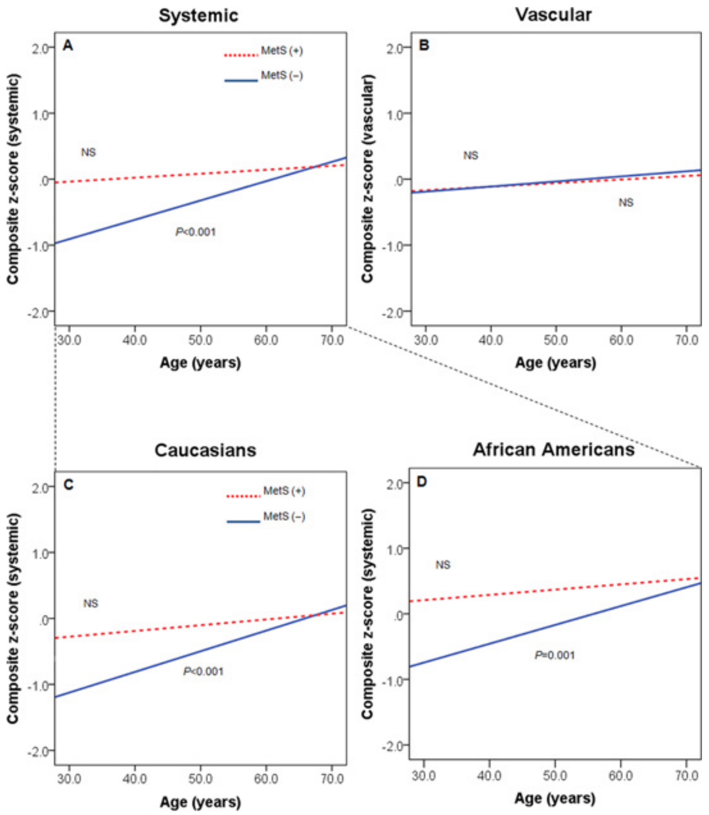

Fig 2. Relationship between age and composite z-score of systemic (**A**) or vascular (**B**) inflammation in all subjects by MetS status.Dashed line represents subjects with MetS, whereas straight line represents subjects without MetS. Relationship between age and composite z-score of systemic inflammation in Caucasians (**C**) and African-Americans (**D**) by MetS status. Lines represent unadjusted linear regression slopes. Dashed line represents subjects with MetS, whereas straight line represents subjects without MetS.

doi:10.1371/journal.pone.0121947.g002

different relationship between age and systemic inflammation depending on the absence or presence of a metabolic burden irrespective of Caucasian/African American ethnicity.

Discussion

Metabolic factors are important predictors of CVD [31]. It is well recognized that a cluster of metabolic abnormalities, defined as MetS including hypertriglyceridemia, low HDL-cholesterol, hypertension, abdominal obesity, and increased fasting glucose levels, has been associated with subsequent development of diabetes mellitus and CVD [31–33]. Presently, according to the NCEP-ATP III definition, roughly one third of middle-aged men and women in the US have MetS, and the frequency increases to above 50% among the elderly [34]. As age is an important CVD risk factor, there is a need to better understand mechanisms underlying the contribution of aging to CVD development.

Increasing studies have indicated that metabolic factors are affected by immunological pro-inflammatory responses. The aging immune microenvironment is characterized as engaging in a systemic, low-grade, chronic inflammatory state that has been termed "inflammaging" [35,36], hallmarked by increased cytokine production, such as IL-6 and TNF-α. Both of these factors induce production of acute phase proteins, including CRP that has become increasingly correlated with cardiovascular risk [37,38]. Moreover, even with healthy aging, a gradual loss of lean body mass results in an overall redistribution of total body mass through increases in

lipid deposition [39]. Similar to the aging process, obesity has been shown to have an immunomodulatory effect, where cross-talk between adipocytes, non-adipose cells, and immune cells is thought to lead towards the development and perpetuation of a "meta-inflammatory" state, defined as a state of chronic low-grade systemic inflammation, currently hypothesized to be responsible for metabolic diseases.

Therefore, in the present study we addressed the issue to what extent an age-associated increase in systemic inflammatory markers would differ depending on the presence of metabolic burden. To test the generalizability of these results, we conducted studies in both humans and mice, in both cases representing a spectrum of age ranges and metabolic conditions. The central novel finding in our study was that the distribution pattern of inflammatory markers over age differed with the presence or absence of a metabolic burden. Our mouse studies confirmed the increase in inflammatory markers, namely acute phase proteins, with age. Yet, we also demonstrated that young (2 month) obese mice with metabolic burden indicated SAA levels that were comparable to their middle-aged (12 month) counterparts. When examining the effects of increased metabolic burden in aged mice, calorie-restriction did not affect SAA levels. In agreement with our mouse studies, human studies demonstrated that levels of inflammatory markers increased significantly with age in subjects without MetS but much less in subjects with MetS, which already reach high levels at an earlier age. The results were similar in our mouse models, representing animals of different ages and weights. Collectively, our results underscore the important influence of a metabolic burden as a modulator for age-related inflammatory response. As our results implicate the presence of an enhanced inflammatory burden already at a younger age in the presence of a metabolic challenge, the findings strengthen the importance of early interventions to reduce conditions favoring CVD development.

A negative metabolic burden present in early life has been suggested to underlie manifestations of chronic disease throughout the lifespan. Childhood obesity is an example of such a metabolic event, and has been suggested to contribute to an alarming shift in the childhood health spectrum from acute to chronic illnesses with long-term negative effects. Furthermore, the presence of the MetS at younger ages is likely to lead to a lifetime inflammatory burden and premature risk for chronic disease manifestations. Although the notion that elevated inflammatory markers increase the risk of CVD has been increasingly recognized [40], underlying mechanisms and pathways remain to be elucidated. Further, an increase of inflammatory cytokines can potentially contribute to the progression of many types of chronic and degenerative diseases beyond atherosclerosis, such as cancer, obesity, diabetes, and congestive heart failure. Thus, presence of these risk factors and CVD itself may in turn further stimulate the inflammatory process, resulting in a vicious cycle. It has been demonstrated that increases in inflammatory markers with age are due, at least partly, to a progressive increase in the burden of cardiovascular risk factor and morbidity [41].

As inflammation is an integral and necessary part of the response to pathogens and constitutes an important part of the immune defense system, it is important to have a better understanding of the role of individual markers of inflammation in predicting risk of disease development. We have previously reported an increase in the systemic, but not vascular, inflammatory burden with age [4], suggesting a more diversified relationship between inflammatory markers and disease. In the present paper, we extended these findings in several ways. First, we extended our study to the impact of a metabolic burden on the relationship between inflammation and age. Second, we sought to assess whether this relationship would be present in an animal model, using mice representing different ages and weight. Our findings in humans and our animal models were similar, implicating that under a metabolic burden, a pro-inflammatory condition was present from an early age, and also that the age-associated increase in inflammatory markers was considerably less. Furthermore, our results

suggest that the presence of the MetS has a greater modulatory effect on age-related inflammatory response for systemic than vascular inflammatory markers. This may implicate that vascular inflammation may reflect more specific to a localized atherogenic process but less to a more widespread inflammatory process.

We acknowledge some of the limitations of this study. In view of the complexity of the inflammatory process and the considerable arsenal of markers available, it is well recognized that a classification of systemic vs. vascular inflammatory markers likely represent a relatively narrow and perhaps somewhat simplistic view. However, we nevertheless adopted this approach as an initial attempt to address the relationship of individual inflammatory markers with other cardiovascular risk factors. Furthermore, the cross-sectional study design does not allow us to evaluate the causative and longitudinal effect of age and other factors that might influence on levels of inflammatory markers. Subjects in our study were recruited from patients scheduled for coronary angiography and are likely more typical of a high-risk patient group than the general population at large. However, utility of high-risk population is particularly valuable in assessing inflammatory factors that might be enriched in this population and ultimately their effects on risk of CVD. We did study two race/ethnicity groups but expanded studies are needed to investigate other ethnic background groups. Although our findings support the notion that systemic inflammatory burden measured as increased levels of inflammatory markers is persistently elevated by age, additional studies are warranted to verify these results in more general populations as well as in prospective studies over the lifespan.

In conclusions, the findings suggest that an increase of inflammatory burden over age differed in subjects with or without a metabolic challenge. Our results underscore the important influence of an unfavorable metabolic status as a modulator for age-related inflammatory response with an enhanced inflammatory burden already at a younger age. The results also emphasize the importance of a better understanding of specific inflammatory pathways in designing preventive measures.

Supporting Information

S1 Table. Pearson's partial (adjusted for gender) correlation coefficients between age and inflammatory markers in subjects with and without MetS across race.
(DOCX)

S1 Fig. Relationship between levels of inflammatory markers and age in subjects with (dotted line, red circles) and without (straight line, blue circles) MetS. A, C-reactive protein (CRP). B, Fibrinogen. C, Serum amyloid-A (SAA). D, Lipoprotein associated phospholipase A2 (Lp-PLA_2) mass; E, Lp-PLA_2 activity. F: Pentraxin-3 (PTX-3). LN indicates logarithmically transformed variables. Lines represent unadjusted linear regression slopes.
(TIF)

Acknowledgments

We thank diaDexus, Inc. for assistance with Lp-PLA_2 measurements.

Author Contributions

Conceived and designed the experiments: AE AM BE WJM LFB. Performed the experiments: AE AM BE WZ WJM LFB. Analyzed the data: AE BE LAB WJM LFB. Contributed reagents/materials/analysis tools: AE AM BE WZ WJM. Wrote the paper: AE AM BE LAB WJM LFB.

References

1. Ross R. Atherosclerosis—an inflammatory disease. N Engl J Med. 1999; 340: 115–126. PMID: 9887164
2. Hansson GK, Hermansson A. The immune system in atherosclerosis. Nat Immunol. 2011; 12: 204–212. doi: 10.1038/ni.2001 PMID: 21321594
3. Libby P, Okamoto Y, Rocha VZ, Folco E. Inflammation in atherosclerosis: transition from theory to practice. Circ J. 2010; 74: 213–220. PMID: 20065609
4. Anuurad E, Enkhmaa B, Gungor Z, Zhang W, Tracy RP, Pearson TA, et al. Age as a modulator of inflammatory cardiovascular risk factors. Arterioscler Thromb Vasc Biol. 2011; 31: 2151–2156. doi: 10.1161/ATVBAHA.111.232348 PMID: 21700927
5. Executive Summary of The Third Report of The National Cholesterol Education Program (NCEP) Expert Panel on Detection, Evaluation, And Treatment of High Blood Cholesterol In Adults (Adult Treatment Panel III). JAMA. 2001; 285: 2486–2497. PMID: 11368702
6. Libby P, Ridker PM, Hansson GK. Progress and challenges in translating the biology of atherosclerosis. Nature. 2011; 473: 317–325. doi: 10.1038/nature10146 PMID: 21593864
7. Flegal KM, Carroll MD, Ogden CL, Curtin LR. Prevalence and trends in obesity among US adults, 1999–2008. JAMA. 2010; 303: 235–241. doi: 10.1001/jama.2009.2014 PMID: 20071471
8. Ervin RB. Prevalence of metabolic syndrome among adults 20 years of age and over, by sex, age, race and ethnicity, and body mass index: United States, 2003–2006. Natl Health Stat Report. 2009: 1–7.
9. Beckett N, Nunes M, Bulpitt C. Is it advantageous to lower cholesterol in the elderly hypertensive? Cardiovasc Drugs Ther. 2000; 14: 397–405. PMID: 10999646
10. Paultre F, Pearson TA, Weil HF, Tuck CH, Myerson M, Rubin J, et al. High levels of Lp(a) with a small apo(a) isoform are associated with coronary artery disease in African American and white men. Arterioscler Thromb Vasc Biol. 2000; 20: 2619–2624. PMID: 11116062
11. Anuurad E, Rubin J, Lu G, Pearson TA, Holleran S, Ramakrishnan R, et al. Protective effect of apolipoprotein E2 on coronary artery disease in African Americans is mediated through lipoprotein cholesterol. J Lipid Res. 2006; 47: 2475–2481. PMID: 16888319
12. Miller M, Mead LA, Kwiterovich PO Jr., Pearson TA. Dyslipidemias with desirable plasma total cholesterol levels and angiographically demonstrated coronary artery disease. Am J Cardiol. 1990; 65: 1–5. PMID: 2294675
13. McGowan MW, Artiss JD, Strandbergh DR, Zak B. A peroxidase-coupled method for the colorimetric determination of serum triglycerides. Clin Chem. 1983; 29: 538–542. PMID: 6825269
14. Allain CC, Poon LS, Chan CS, Richmond W, Fu PC. Enzymatic determination of total serum cholesterol. Clin Chem. 1974; 20: 470–475. PMID: 4818200
15. Warnick GR, Benderson J, Albers JJ. Dextran sulfate-Mg2+ precipitation procedure for quantitation of high-density-lipoprotein cholesterol. Clin Chem. 1982; 28: 1379–1388. PMID: 7074948
16. Friedewald WT, Levy RI, Fredrickson DS. Estimation of the concentration of low-density lipoprotein cholesterol in plasma, without use of the preparative ultracentrifuge. Clin Chem. 1972; 18: 499–502. PMID: 4337382
17. Macy EM, Hayes TE, Tracy RP. Variability in the measurement of C-reactive protein in healthy subjects: implications for reference intervals and epidemiological applications. Clin Chem. 1997; 43: 52–58. PMID: 8990222
18. Ridker PM, Cushman M, Stampfer MJ, Tracy RP, Hennekens CH. Inflammation, aspirin, and the risk of cardiovascular disease in apparently healthy men. N Engl J Med. 1997; 336: 973–979. PMID: 9077376
19. Clauss A. [Rapid physiological coagulation method in determination of fibrinogen.]. Acta Haematol. 1957; 17: 237–246. PMID: 13434757
20. Adler AI, Levy JC, Matthews DR, Stratton IM, Hines G, Holman RR. Insulin sensitivity at diagnosis of Type 2 diabetes is not associated with subsequent cardiovascular disease (UKPDS 67). Diabet Med. 2005; 22: 306–311. PMID: 15717879
21. Enkhmaa B, Anuurad E, Ozturk Z, Zhang W, Pearson TA, Berglund L. Differential associations of serum amyloid A and pentraxin-3 with allele-specific lipoprotein(a) levels in African Americans and Caucasians. Transl Res. 2011; 158: 92–98. doi: 10.1016/j.trsl.2011.01.004 PMID: 21757153
22. Dada N, Kim NW, Wolfert RL. Lp-PLA2: an emerging biomarker of coronary heart disease. Expert Rev Mol Diagn. 2002; 2: 17–22. PMID: 11963798
23. Koenig W, Twardella D, Brenner H, Rothenbacher D. Lipoprotein-associated phospholipase A2 predicts future cardiovascular events in patients with coronary heart disease independently of traditional risk factors, markers of inflammation, renal function, and hemodynamic stress. Arterioscler Thromb Vasc Biol. 2006; 26: 1586–1593. PMID: 16627803

24. Kosaka T, Yamaguchi M, Soda Y, Kishimoto T, Tago A, Toyosato M, et al. Spectrophotometric assay for serum platelet-activating factor acetylhydrolase activity. Clin Chim Acta. 2000; 296: 151–161. PMID: 10807978

25. Grundy SM, Cleeman JI, Daniels SR, Donato KA, Eckel RH, Franklin BA, et al. Diagnosis and management of the metabolic syndrome: an American Heart Association/National Heart, Lung, and Blood Institute Scientific Statement. Circulation. 2005; 112: 2735–2752. PMID: 16157765

26. Anuurad E, Ozturk Z, Enkhmaa B, Pearson TA, Berglund L. Association of lipoprotein-associated phospholipase A2 with coronary artery disease in African-Americans and Caucasians. J Clin Endocrinol Metab. 2010; 95: 2376–2383. doi: 10.1210/jc.2009-2498 PMID: 20194707

27. Anuurad E, Tracy RP, Pearson TA, Kim K, Berglund L. Synergistic role of inflammation and insulin resistance as coronary artery disease risk factors in African Americans and Caucasians. Atherosclerosis. 2009; 205: 290–295. doi: 10.1016/j.atherosclerosis.2008.11.028 PMID: 19135196

28. Borkan GA, Hults DE, Gerzof SG, Robbins AH. Comparison of body composition in middle-aged and elderly males using computed tomography. Am J Phys Anthropol. 1985; 66: 289–295. PMID: 3985138

29. Gallagher D, Ruts E, Visser M, Heshka S, Baumgartner RN, Wang J, et al. Weight stability masks sarcopenia in elderly men and women. Am J Physiol Endocrinol Metab. 2000; 279: E366–375. PMID: 10913037

30. Goodpaster BH, Kelley DE, Thaete FL, He J, Ross R. Skeletal muscle attenuation determined by computed tomography is associated with skeletal muscle lipid content. J Appl Physiol (1985). 2000; 89: 104–110.

31. Reaven GM. Banting lecture 1988. Role of insulin resistance in human disease. Diabetes. 1988; 37: 1595–1607. PMID: 3056758

32. Grundy SM. Does a diagnosis of metabolic syndrome have value in clinical practice? Am J Clin Nutr. 2006; 83: 1248–1251. PMID: 16762931

33. Reaven GM. The metabolic syndrome: is this diagnosis necessary? Am J Clin Nutr. 2006; 83: 1237–1247. PMID: 16762930

34. Ford ES, Giles WH, Dietz WH. Prevalence of the metabolic syndrome among US adults: findings from the third National Health and Nutrition Examination Survey. JAMA. 2002; 287: 356–359. PMID: 11790215

35. Giunta S. Is inflammaging an auto[innate]immunity subclinical syndrome? Immun Ageing. 2006; 3: 12. PMID: 17173699

36. Franceschi C. Inflammaging as a major characteristic of old people: can it be prevented or cured? Nutr Rev. 2007; 65: S173–176. PMID: 18240544

37. Mendall MA, Patel P, Asante M, Ballam L, Morris J, Strachan DP, et al. Relation of serum cytokine concentrations to cardiovascular risk factors and coronary heart disease. Heart. 1997; 78: 273–277. PMID: 9391290

38. Miller AM, McInnes IB. Cytokines as therapeutic targets to reduce cardiovascular risk in chronic inflammation. Curr Pharm Des. 2011; 17: 1–8. PMID: 21222645

39. Guo SS, Zeller C, Chumlea WC, Siervogel RM. Aging, body composition, and lifestyle: the Fels Longitudinal Study. Am J Clin Nutr. 1999; 70: 405–411. PMID: 10479203

40. Tracy RP, Lemaitre RN, Psaty BM, Ives DG, Evans RW, Cushman M, et al. Relationship of C-reactive protein to risk of cardiovascular disease in the elderly. Results from the Cardiovascular Health Study and the Rural Health Promotion Project. Arterioscler Thromb Vasc Biol. 1997; 17: 1121–1127. PMID: 9194763

41. Ferrucci L, Corsi A, Lauretani F, Bandinelli S, Bartali B, Taub DD, et al. The origins of age-related proinflammatory state. Blood. 2005; 105: 2294–2299. PMID: 15572589
